# Mechanistic insight into hyaluronic acid and platelet-rich plasma-mediated anti-inflammatory and anti-apoptotic activities in osteoarthritic mice

**DOI:** 10.18632/aging.101713

**Published:** 2018-12-23

**Authors:** Chi-Sheng Chiou, Chi-Ming Wu, Navneet Kumar Dubey, Wen-Cheng Lo, Feng-Chou Tsai, Tran Dang Xuan Tung, Wei-Ching Hung, Wei-Che Hsu, Wei-Hong Chen, Win-Ping Deng

**Affiliations:** 1 School of Dentistry, College of Oral Medicine, Taipei Medical University, Taipei, Taiwan; 2 Division of Allergy, Immunology and Rheumatology, Department of Internal Medicine, Taipei Medical University Hospital, Taipei, Taiwan; 3 Graduate Institute of Biomedical Materials and Tissue Engineering, College of Biomedical Engineering, Taipei Medical University, Taipei, Taiwan; 4 Ceramics and Biomaterials Research Group, Advanced Institute of Materials Science, Ton Duc Thang University, Ho Chi Minh City, Vietnam; 5 Faculty of Applied Sciences, Ton Duc Thang University, Ho Chi Minh City, Vietnam; 6 School of Medicine, College of Medicine, Taipei Medical University, Taipei, Taiwan; 7 Department of Neurosurgery, Taipei Medical University Hospital, Taipei, Taiwan; 8 School of Dentistry, College of Oral Medicine, Taipei Medical University, Taipei, Taiwan; 9 Stem Cells Center, Van Hanh General Hospital, Ho Chi Minh City, Vietnam; 10 Stem Cell Research Center, College of Oral Medicine, Taipei Medical University, Taiwan; 11 Graduate Institute of Basic Medicine, Fu Jen Catholic University, Taipei, Taiwan

**Keywords:** hyaluronic acid, platelet-rich plasma, osteoarthritis, chondrocyte, inflammation, apoptosis

## Abstract

Osteoarthritis (OA) poses a major clinical challenges owing to limited regenerative ability of diseased or traumatized chondrocytes in articular cartilage. Previous studies have determined the individual therapeutic efficacies of hyaluronic acid (HA) and platelet-rich plasma (PRP) on OA; however, the underlying mechanism is still lacking. Therefore, we investigated mechanistic approach of HA+PRP therapy on chondrocyte apoptosis in IL-1β+TNF-α (I+T) treated *in vitro* OA model, in addition to *in vivo* anterior cruciate ligament transection-OA mice model. MTT assay showed an enhanced chondrocyte proliferation and viability in HA+PRP-treated group, compared to I+T, I+T/HA, I+T/PRP, I+T/HA+PRP groups. Further, HA+PRP also significantly suppressed ROS, apoptotic cleaved caspase-3 and PARP, p53 and p21 and MMP-1; whereas, cell cycle modulatory proteins including p-ERK, cyclin B1, D1, and E2 were upregulated. The sub-G1 population and TUNEL assay confirmed the higher abundance of healthy chondrocytes in HA+PRP group. A significantly decreased ARS staining in HA+PRP group was also noted, indicating reduced cartilaginous matrix mineralization compared to other groups. Conclusively, compared to HA or PRP, the combined HA+PRP might be a promising therapy for articular cartilage regeneration in osteoarthritic pathology, possibly via augmented anti-inflammatory, anti-oxidative chondrocyte proliferation and inhibited MMP-1 activity and matrix calcification.

## Introduction

Osteoarthritis (OA) is the debilitating joint condition distinguished by progressive degradation of articular cartilage, leading to stiffness, excessive pain and crepitus. This is primarily attributed to degenerating chondrocytes, which are the sole cell type responsible for synthesizing and mantaining the extracellular matrix [1]. A growing body of evidence demonstrate that the chondrocyte apoptosis plays a key role in OA physiopathology in which the hyaline articular cartilage covering the articular surfaces undergo mild to severe degradation [2–4]. This has specifically been attributed to enhanced mechanical and inflammatory stresses; additionally, the limited regenerative ability of articular chondrocytes, owing to low mitotic activity renders them highly susceptible to damage leading to reduced ECM deposition and imbalanced knee-joint homeostasis [5,6]. It has been forecasted that by the year 2020, over 25% of adult population will be affected by OA, and would be a leading cause of morbidity among individuals over the age of 40 [7]. Currently, apart from pain management and surgical intervention, no effective disease-modifying therapy exists for OA; hence, the novel treatment method is urgently needed. The hyaluronic acid (HA), a natural disaccharide polymer can mimic the synovial fluid to support joint lubrication and maintain the chondrocyte functions and ECM synthesis through inhibiting the inflammatory process [8]. Besides, the platelet-rich plasma (PRP) containing plethora of growth factors has shown promising results in regenerating cartilage, which is majorly attributed to TGF-β1 and platelet-derived grow factor (PDGF), stimulating chondrocyte proliferation and proteoglycan biosynthesis [2]. A few previous studies have evaluated the synergistic therapeutic efficacies of HA and PRP on OA; however, the detailed mechanism involved is still lacking [2,3]. Therefore, we aimed to determine the mechanistic insight underlying the augmented anabolic activities of HA+PRP, particularly through suppression of apoptosis. Initially, the HA+PRP was administered to osteoarthritic chondrocytes *in vitro* and further in the knee-joint of anterior cruciate ligament transection (ACLT)-induced OA mouse model. We simulated the inflammatory osteoarthritic microenvironment in articular chondrocytes by using pro-inflammatory cytokines, the interleukin-1β (IL-1β) and tumor necrosis factor-α (TNF-α), which participate in catabolic degradation of ECM proteins.

Further, it has been demonstrated that chondrocyte apoptosis caused by cytokines may be induced by various signals, such as caspase-3 and reactive oxygen species (ROS) [9,10]. Furthermore, the proteolytic activities of accumulated matrix metalloproteinase (MMPs) are known to degrade ECM of articular cartilage [11]. Hence, we investigated the levels of MMP-1 in the tissues of OA knee-joint. On the other hand, the chondrocyte hypertrophy and matrix mineralization in OA cartilage occurs near sites of injury [12]. Therefore, the effect of HA+PRP on presence of calcium deposits in chondrocytes-mediated synthesis of ECM was also detected. Conclusively, this study will provide the mechanistic basis of HA+PRP treatment in *in vitro* and *in vivo* OA model.

## RESULTS

### Combinational effect of HA+PRP on proliferation and viability of chondrocytes

Cartilage regeneration is accompanied by several factors in which inhibition of apoptosis plays an important role. Hence, we investigated anti-apoptotic mechanism mediated by HA+PRP in the chondrocytes obtained from osteoarthritic patients.

**Figure 1:**
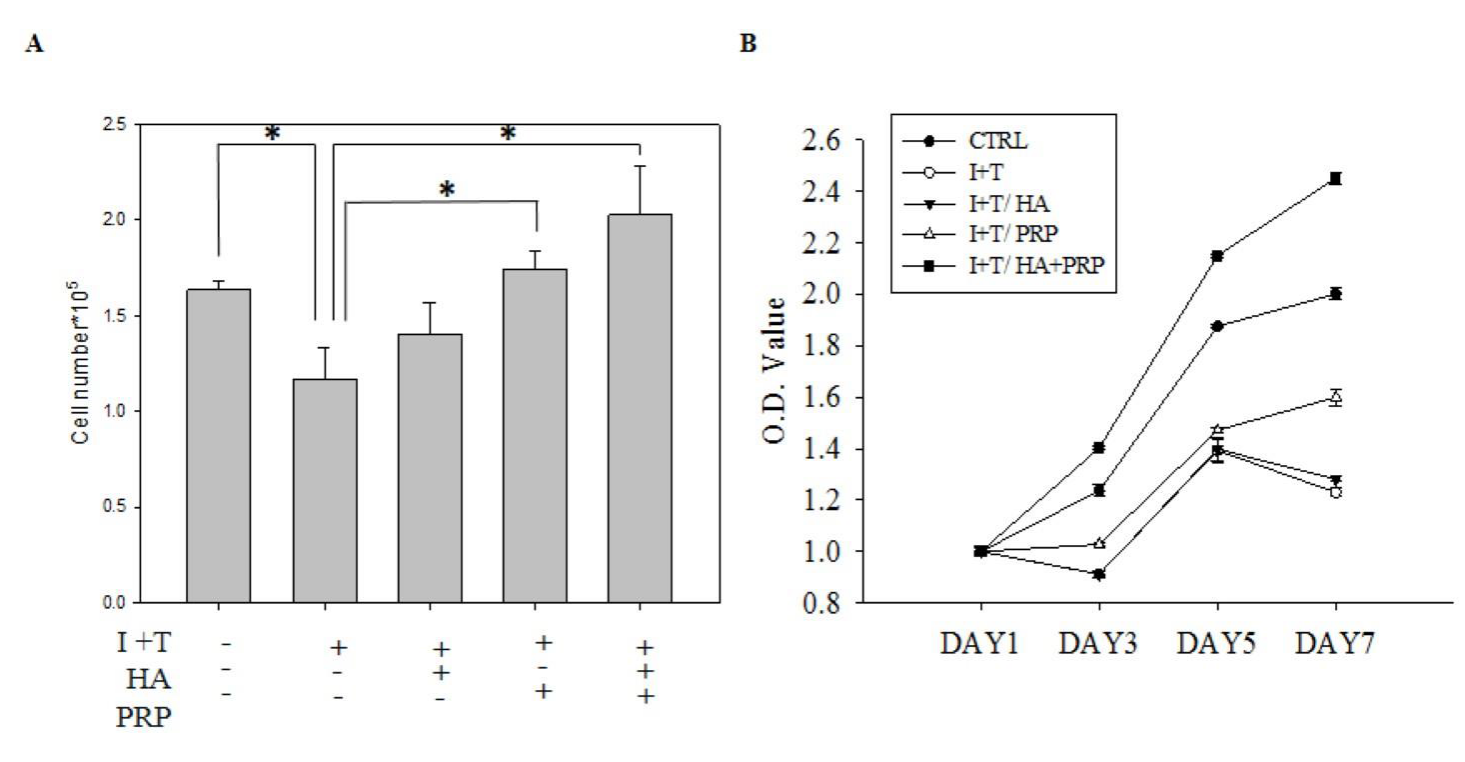
**Effects of platelet-rich plasma and hyaluronic acid (HA+PRP) on cellular activity of primary chondrocytes obtained from osteoarthritic patients.** (**A**) *In vitro* proliferation ability of chondrocytes was examined after two-day treatment of IL-1β+ TNF-α (I+T) conditioned medium in the presence of HA, PRP, and HA+PRP. (**B**) Assessment of cell viability on day 1, 3, 5, and 7 via MTT assay in HA, PRP, and HA+PRP treated chondrocytes. CTRL, control; I, IL-1β; T, TNF-α. *p<0.01, compared with the value in cells cultured in I+T using student t-test. The results are presented as mean ± S.D. for 15 independent experimental replicates.

To determine the synergistic effect of HA and PRP (HA+PRP), the cell numbers and extent of viability of chondrocytes were assessed after treatment with IL-1β+ TNF-α (I+T) for 2 days (Figure 1A). Chondrocyte treated by I+T demonstrated a significantly reduced cell numbers (1.167 ± 0.165 vs. CTRL: 1.633 ± 0.047), which were further restored by HA (1.402 ± 0.166), PRP (1.74 ± 0.099), and particularly by HA+PRP (2.027 ± 0.253 vs. CTRL). Moreover, the cell viability of chondrocytes was investigated by MTT assay (Figure 1B). At day 7, the higher absorbance values of HA+PRP-treated group (2.4517 ± 0.0235) demonstrated a very positive effect on the viability of chondrocytes inhibited by I+T when compared to HA (1.281 ± 0.099), PRP (1.5995 ± 0.033), and CTRL (2.0012 ± 0.021; vs. CTRL). However, HA+PRP treatment diminished expression of apoptotic proteins in chondrocyte.

**Figure 2:**
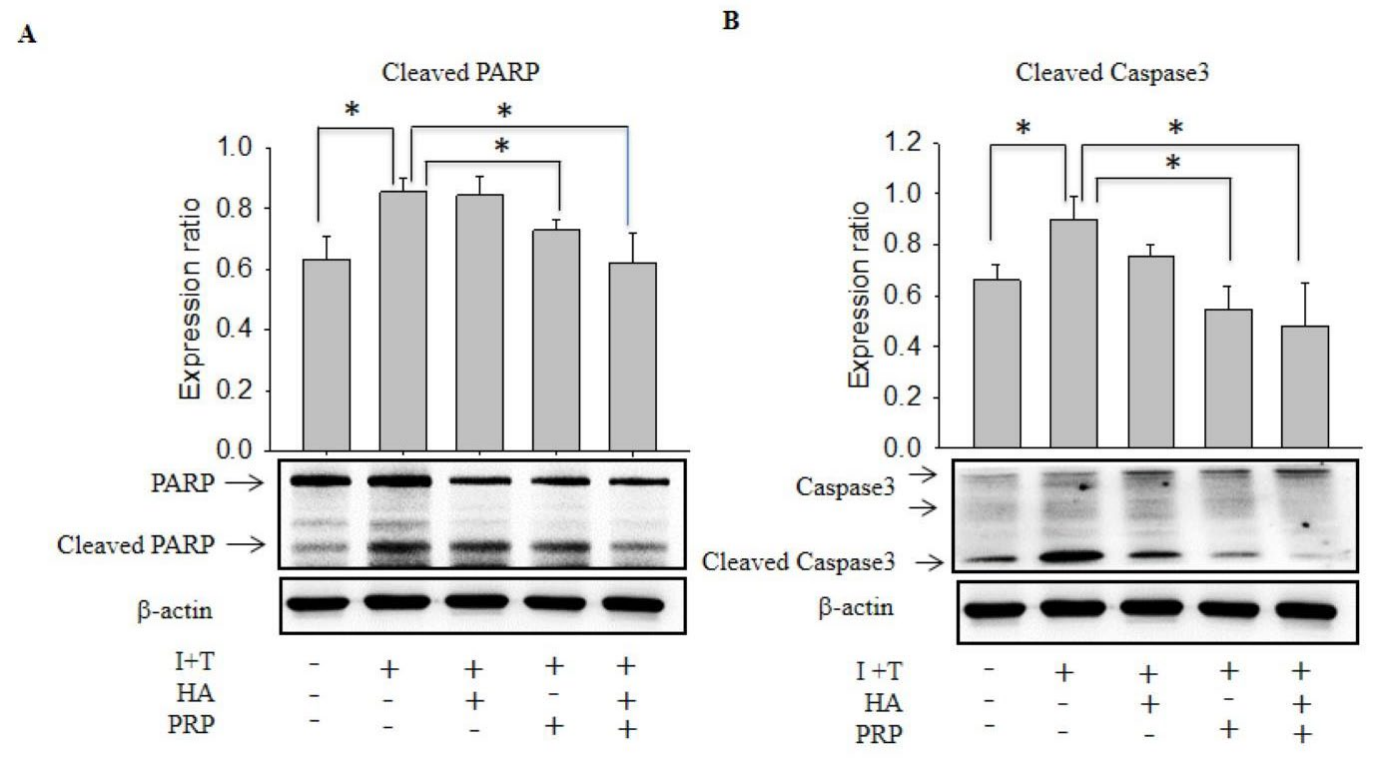
**Effects of HA+PRP on inhibition of cellular apoptosis-related proteins in chondrocytes.** Western blot analysis of (**A**) cleaved PARP and (**B**) cleaved caspase-3 after treatment of I+T conditioned medium in the presence of HA, PRP, and HA+PRP. *p<0.05, compared with the value in cells cultured in I+T using student t-test. The results are presented as mean ± S.D. for 15 independent experimental replicates.

Cleaved caspase-3 and cleaved PARP are thought to play a key role in cellular apoptosis [13], which are activated in inflammatory microenvironment. Therefore, we investigated the release of these apoptotic proteins via chondrocytes by western blot. The I+T group demonstrated a significantly increased expression of cleaved Caspase-3 and Cleaved PARP (Cleaved Caspase-3: 0.897 ± 0.099 vs. CTRL: 0.6617 ± 0.062; Cleaved PARP 0.856 ± 0.045 vs. CTRL 0.631 ± 0.076), which were further decreased by PRP (Cleaved Caspase-3: 0.547 ± 0.099; Cleaved PARP 0.728 ± 0.37). Notably, an obvious decline was found in HA+PRP group (Cleaved Caspase-3: 0.48 ± 0169; Cleaved PARP 0.620 ± 0.098) (Figure 2A &B, respectively).

### HA+PRP treatment and apoptotic signaling

**Figure 3:**
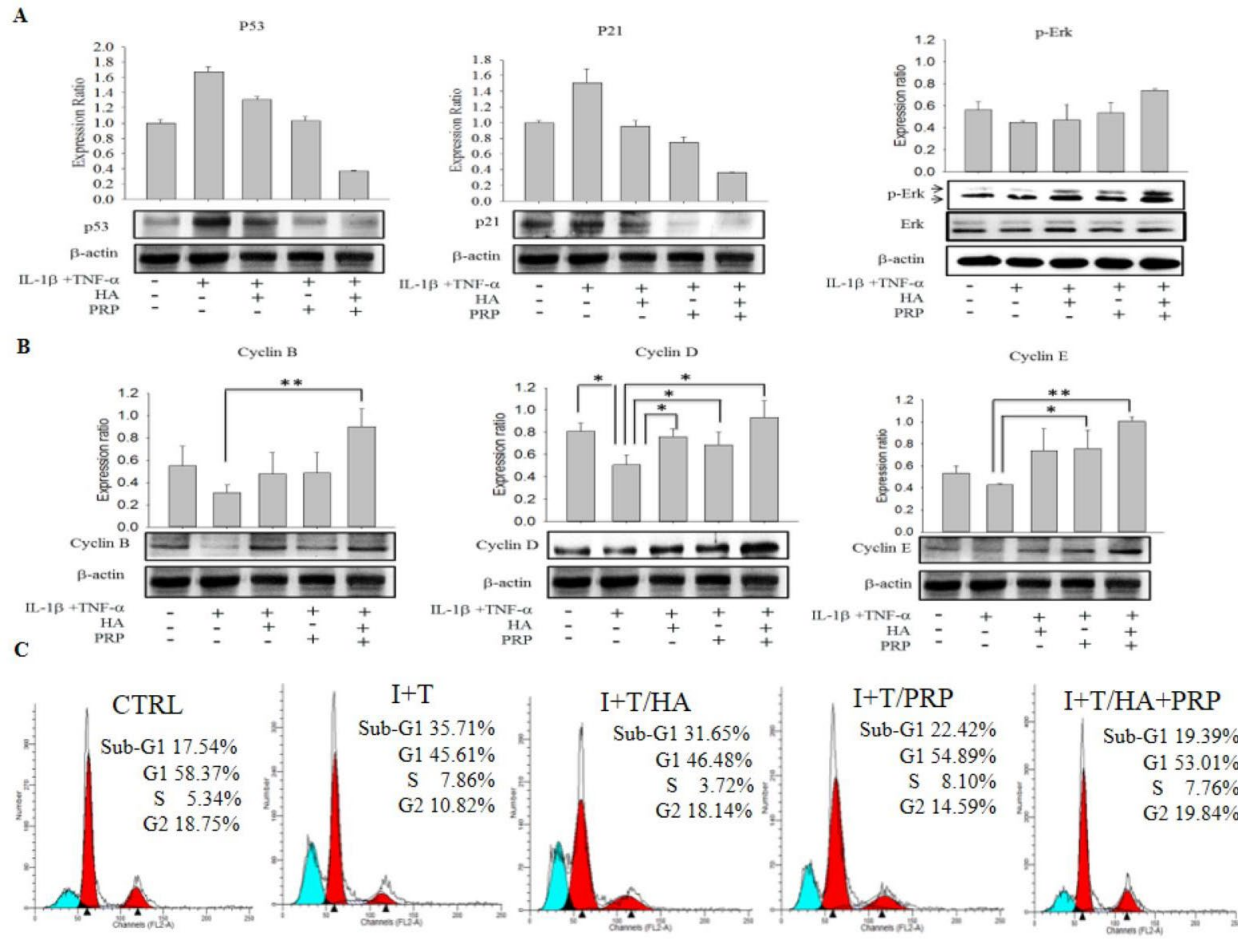
**Efficacy of HA+PRP treatment on apoptotic signaling and cell cycle modulatory proteins in chondrocytes. (A**) Expression of p53, p21 and p-ERK were assessed by western blot after treatment in I+T conditioned medium in the presence of HA, PRP, and HA+PRP. (**B**) cyclin B, cyclin D, and cyclin E were assessed by western blot after treatment of I+T conditioned medium in the presence of HA, PRP, and HA+PRP. (**C**) Cell cycles distributions of chondrocytes. Percentages of sub-G1, G1, S, and G2 phases recorded by flow cytometry after treatment of I+T conditioned medium in the presence of HA, PRP, and HA+PRP. *p<0.05, **P<0.01, compared with the value in cells cultured in I+T using student *t-test*. The results are presented as mean ± S.D. for 15 independent experimental replicates.

p53 is an identified regulatory protein that participate in signaling pathway and recruits an array of biochemical activities to trigger diverse biologic responses, most notably cell cycle arrest and apoptosis via expression of p21 protein [14,15]. In our study, the western blot results showed an increased expression of p53 and p21 in I+T group, which were highly diminished in HA+ PRP treated group (Figure 3A, p53 and p21, respectively). Further, the expression of cell cycle modulatory proteins including p-ERK, cyclin B1, D1, and E2 were investigated, which were found to be significantly enhanced in HA+PRP treated group as compared to I+T group (Figure 3A, p-ERK; Figure 3B, cyclin B1, D1 and E2, respectively).

### Effect of HA+PRP treatment on cell cycle arrest

The presence of apoptotic cells manifests as the “sub-G1” fractions of cell cycle [16]. These apoptotic cell population was analyzed during cell cycle phase distribution using flow cytometric analysis. The data revealed that the percentages of cellular population in sub-G1 phases, was higher in I+T group (35.71%) compared to I+T/HA (31.65%), I+T/PRP (22.42%), which was effectively decreased through the treatment of HA+PRP (19.39%) and found comparable to control (17.54%) (Figure 3C). This data indicates that HA+PRP treatment reversed the cell cycle arrest in sub-G1 phase.

### Influence of HA+PRP on oxidative stress

**Figure 4:**
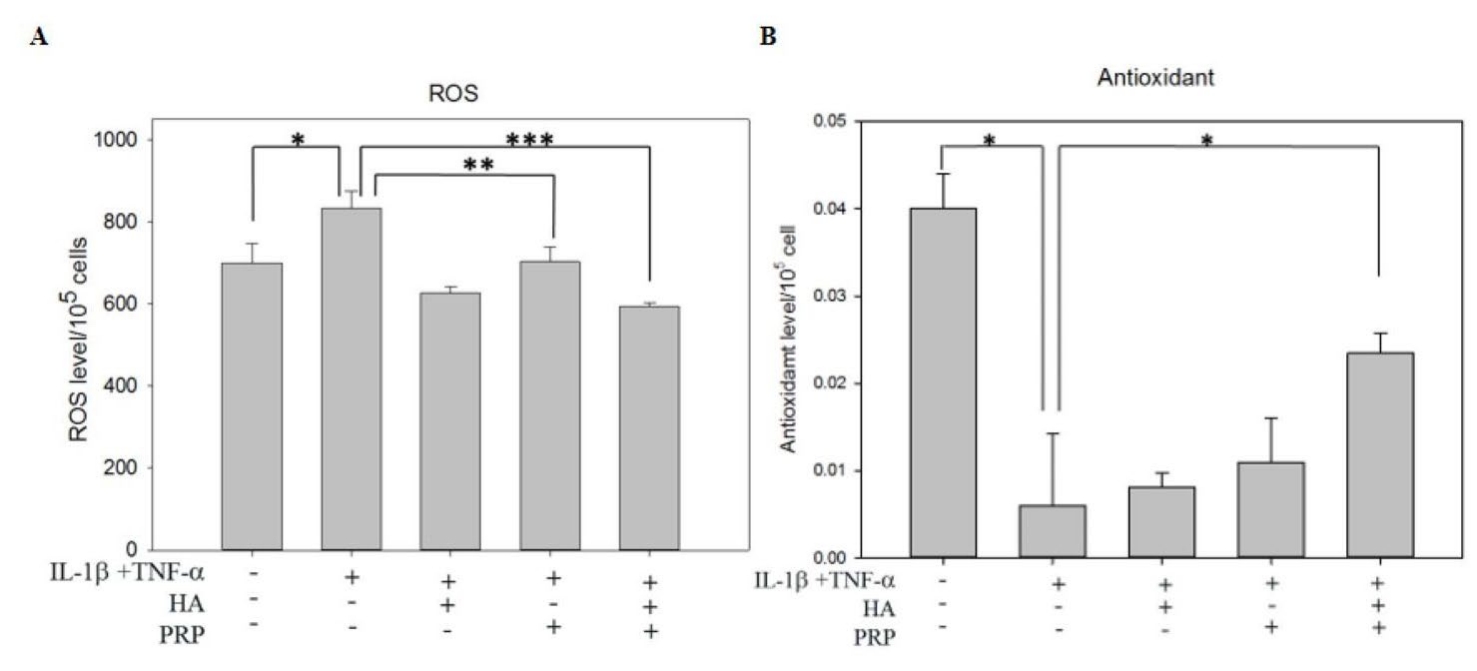
**Effects of HA+PRP on oxidative stress in chondrocytes. (A**) ROS and (**B**) antioxidant levels of chondrocytes were recorded after treatment of IL-1β+TNF-α (I+T) conditioned medium in the presence of HA, PRP, and HA+PRP. *p<0.05, **p<0.01, ***p<0.001, compared with the value in cells cultured in I+T using student t-test. The results are presented as mean ± S.D. for 15 independent experimental replicates.

ROS plays pivotal role in the oxidative damage in chondrocytes [17]. ROS levels were increased in I+T treated groups (834.33 ± 41.292 vs. CTRL: 699.98 ± 47.33) and were decreased mildly in PRP treated groups (703.33 ± 36.18) and were strongly in HA+PRP group (594.32 ± 8.41) (Figure 4A). On the other hand, the levels of antioxidant were measured, which showed an obvious decrease in I+T treated group (0.00598 ± 0.0082 vs. CTRL: 0.0401 ± 0.0039). No significant changes were recorded in HA or PRP treated groups; however, an increased anti-oxidant level was noted in HA+PRP treated group (0.0235 ± 0.00226) (Figure 4B). The overall result indicated that HA+PRP treatment might suppress ROS levels through increased anti-oxidative activity.

### Effect of HA+PRP on knee-joint ultrastructure

**Figure 5:**
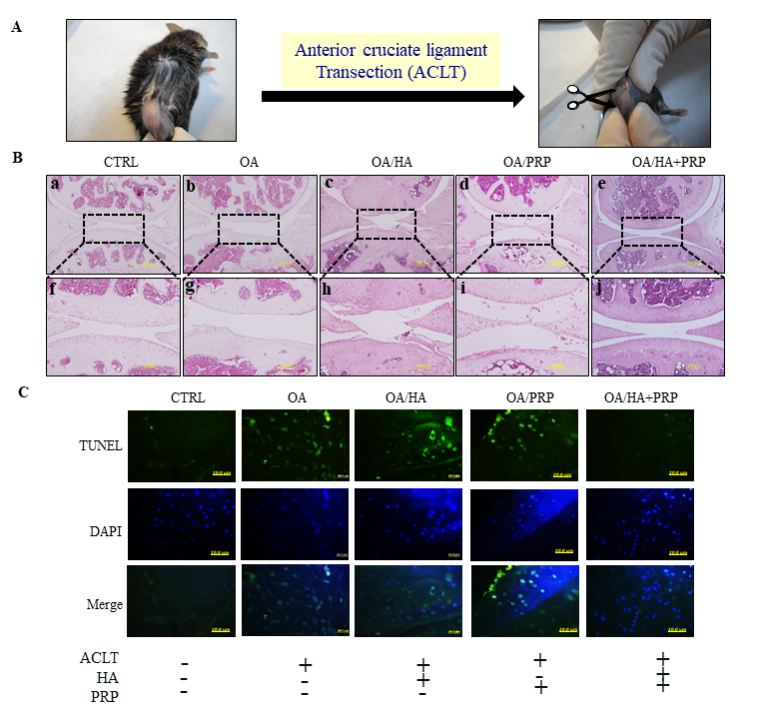
**TUNEL labeling of anterior cruciate ligament transection (ACLT) induced osteoarthritic right knee of 8-months old female C57BL/6J mice.** (**A**) The anterior cruciate ligament of knee-joint was removed through surgical knife and OA was established after 1 month. The HA, PRP, and HA+PRP were injected into right knee after establishment of ACLT-OA for one month. After sacrifice, the right knees biopsies were collected for TUNEL assay to detect cellular apoptosis. (**B**) Histologic hemotoxylin and eosin (H&E)-stained sections revealing ultrastructural architecture of articular cartilage in knee joint of control, OA, HA, PRP and HA+PRP group. The upper panel (a-e, respectively) represent lower magnification (500µm). The dotted rectangular areas in lower magnification images are shown as higher magnification (200 µm) (f-j, respectively). (**C**) Representative images of TUNEL staining in the knee joint after cartilage green staining indicating TUNEL positive (apoptotic) cells against a DAPI (blue) nuclear counter stain.

Further, the therapeutic efficacies of HA+PRP were examined in *in vivo* OA model established through ACLT surgical process (Figure 5A). Hematoxylin and eosin-stained section revealed that control group revealed normal appearing articular cartilage in which the articular surface found to be intact and smooth (Figure 5B; a and f). The meniscus, territorial and inter-territorial matrices were well-organized representing healthy cartilage. On the other hand, OA group showed the presence of clefts on articular surfaces, fibrillation and erosion through cartilage zones (Figure 5B; b and g). Loosened plexus of wavy collagen fibrils with a greater loss of chondrocytes in the extracellular matrix were identified. Upon treatment with HA (Figure 5B; c and h) or PRP (Figure 5B; d and i), little improvements were observed; however, the recovery in combined treatment of HA+PRP (Fig. 3B, e and j) was comparable to control, indicating a significant attenuation of OA.

### TUNEL labeling-dependent apoptotic chondrocyte detection

TUNEL assay was used to detect chondrocytes undergoing extensive DNA degradation during the late stages of apoptosis [18]. A highly TUNEL positive signals were detected in ACLT group compared to control, indicating enhanced apoptotic events; however, the decreased signals were observed in HA or PRP groups, particularly higher in HA+PRP treated group (Figure 5C).

### Histologic evaluation of HA+PRP on matrix degradation and mineralization in hypertrophic chondrocytes

**Figure 6:**
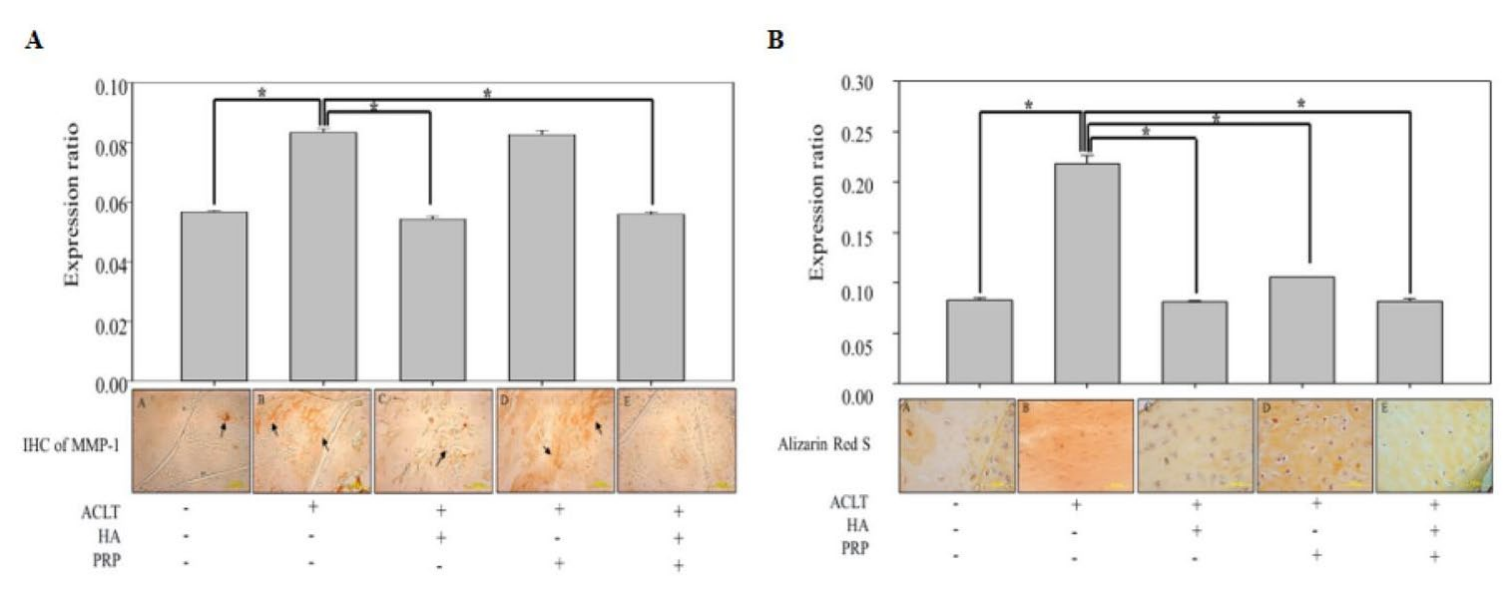
***In vivo* anti-inflammatory and anti-hypertrophic efficacy of HA+PRP in ACLT-OA knee joint.** (**A**) Immunohistochemical (IHC) staining for matrix metalloproteinase (MMP-1) distributions (indicated by black arrows) and their relative quantification in ACLT, HA, PRP and HA+PRP treated groups (**B**) Alizarin Red S staining and their relative quantification for detection of calcium depositions (red color), indicating chondrocyte hypertrophy in ACLT, HA, PRP and HA+PRP treated groups. *p<0.05, The results are presented as mean ± S.D. for 9 independent experimental replicates.

The biological roles of MMPs has traditionally been associated with apoptosis and degradation, and turnover of most of the constituents of the cartilaginous extracellular matrix [18]. Therefore, we conducted immunohistochemical (IHC) staining of MMP-1 in ACLT-OA knee-joint sections, which demonstrated intense brown colored matrix in OA, HA and PRP-treated group, while a feeble staining was observed in HA+PRP-treated group (Figure 6A). Besides, mineralization is also crucial for the development and function of other mineralized tissues; however, excessive mineralization participate in loss of elasticity and resilience of normal tissue [19]. So, we conducted alizarin red S staining to assess the presence of mineralization due to calcium deposition. A higher red colored calcium deposits were observed in OA groups, compared with control group, and those were increased more in PRP treatment, however were decreased in HA, particularly in HA+PRP groups (Figure 6B). These results indicated that the combined treatment of HA+PRP group suppressed the cartilaginous matrix degradation and chondrocytic hypertrophy.

## DISCUSSION

In the clinics, the use of HA or PRP is nowadays increasing for OA therapy, due to limited efficacy of currently available treatments, which only relieve symptoms, such as knee pain, stiffness and swellings [20]. A few studies have utilized HA+ PRP to demonstrate the synergistic anabolic actions on cartilage regeneration [2,3], which is accompanied by several factors in which inhibition of apoptosis plays an important role. In this study, we employed IL-1β+TNF-α (I+T), the well-known inflammatory cytokines, which in synergism exert the degenerative pathologic conditions [3,21], thereby leading to articular cartilage degradation [22]. The enhanced cell number and viability after HA+PRP treatment indicated their anti-inflammatory and chondrocyte-proliferation activity, which is in concord with recent study reporting the anti-inflammatory and wound healing potential of HA in the surgical-site infections [23]. Further, contained with plethora of growth factors particularly TGF-β1, the PRP also seems to be chondroprotective through activation of the anabolic signal pathway, leading to re-synthesis of cartilage through suppressing inflammatory activities [3]. HA is present as side chain in aggrecan and form HA-aggrecan complex, which compounds with water and type II collagen to form extracellular matrix (ECM) [24]. This chondrocytes metabolism and ECM could be degraded by protease activities of MMP through induction of inflammatory response [8,25,26].

Besides, the cleaved caspase-3 and cleaved PARP are proteins known to mediate in cellular apoptosis [27,28]. Previous studies have demonstrated that HA and PRP might activate mitogen-activated protein kinases (MAPKs) ERK pathway and enhance cellular mitoses [29,30]. HA can modify mitochondria to produce ATPs to increase cellular activities [31]. In agreement to these reports, our data also showed the suppressed levels of cleaved caspase-3 and cleaved PARP proteins by HA+PRP treatment, indicating anti-apoptotic activities.

Furthermore, the activated p53 and p21 and inhibited cyclin B, D and E have been reported to adversely influence the cell cycle leading to its arrest [32,33]. Our western blot results also revealed that the combined HA+PRP treatment resulted in cellular apoptosis through modulating ERK pathway and inhibited expression of p53 protein [27,34]. Besides, the TGF-β1 contained in PRP can phosphorylate Smad2/3 to activate cyclin D pathway, thereby inhibit the cell cycle arrest. Cyclin D is the check point modulatory protein from G0 phase to G1 phase, which when inhibited render the cell cycle to enter into sub-G1 phase [35]. After HA+PRP treatment, the level of cyclin D was increased resulting in decreased percentage of cells in sub-G1 phase by activating ERK and inhibiting p53 and p21 proteins.

The ROS is considered as mediator of p53- and p21-dependent apoptosis [36,37] through imparting oxidative stress and suppressing the level of anti-oxidants [38]. Nevertheless, previous studies have demonstrated that HA is a better anti-oxidant and repress ROS and p53 levels [39–41], and also modulate ERK pathway to inhibit apoptosis [27,34]. In concord with these data, we detected a higher level of ROS in OA group implying cellular damage, which were later decreased after HA+PRP treatment. These results indicated the anti-oxidative activities of HA+PRP, which was further confirmed via marked increase in elevated antioxidant level. Interestingly, the insulin-like growth factor (IGF) contained in PRP might have induced anti-oxidative activities via inhibiting release of ROS [42]. These data support our results showing a significantly suppressed levels of ROS through synergistic anti-oxidative actions of HA+PRP.

After confirming the chondrocyte proliferation and anti-inflammatory activities of HA+PRP *in vitro*, we further explored their *in vivo* therapeutic efficacy in the ACLT-induced OA mice model, which closely resembles to human osteoarthritic pathology [43]. As revealed by H&E staining, the impaired histomorphological architecture of articular tissues, were indicative hallmarks of osteoarthritis showing compromised knee-joints, which were restored by HA+PRP treatment. For apoptotic cells, we conducted immunohistochemical TUNEL assay to detect the DNA fragmentation, which is done through labelling of DNA by terminal deoxynucleotidyl transferase enzyme, and could be recognized by green fluorescence [44].

In our study, a highly decreased TUNEL signals in HA+PRP treated group indicated lower apoptotic events and enhanced chondrocyte survival, compared to other groups. Besides, the IL-1β and TNF-α have been reported to stimulate the synthesis of matrix-degrading enzymes like MMPs in OA [45,46]. The matrix MMP-1, a kind of collagenase degrading collagen type I, II and III [22,47,48], play an important role in destruction cartilage leading to OA [49,50]. We found a notable decrease in magnitude of MMP-1 in HA group, which is supported by a few seminal studies demonstrating that hyaluronan with high molecular weight might inhibit inflammatory activity of MMPs [51–53]. The insulin-like growth factor-1 (IGF-1) present in PRP may also inhibit adverse effects of MMP-1 [54]. On contrary, the TGF-β1 and PDGF contained in PRP have been documented to increase the level of MMP-1 [55], which was also observed in PRP-treated group. However, in concord to TUNEL assay data, a significantly suppressed MMP-1 level was exhibited in the blend of HA+PRP treatment, which imply that the inhibitory effect against MMP-1, dominates in the concerted treatment. OA initiates from the articular surface leading to chondrocytes proliferation and aggregation to form hypertrophic chondrocytes in calcified cartilage [56]. Collagens and proteoglycans productions are also decreased in these hypertrophic chondrocytes [57], thereby triggering the process of apoptosis. Therefore, we conducted alizarin red staining to detect calcified matrix. Our results revealed a decreased staining in HA group, which might be attributed to inhibition of MMP-1. However, an increased staining in PRP group, may be due to increased effect of MMP-1 inducing osteocytes response and leading to chondrocyte calcification [58]. However, the staining was very feeble in HA+PRP treated group, indicating anti-inflammatory and anti-hypertrophic activity.

**Figure 7:**
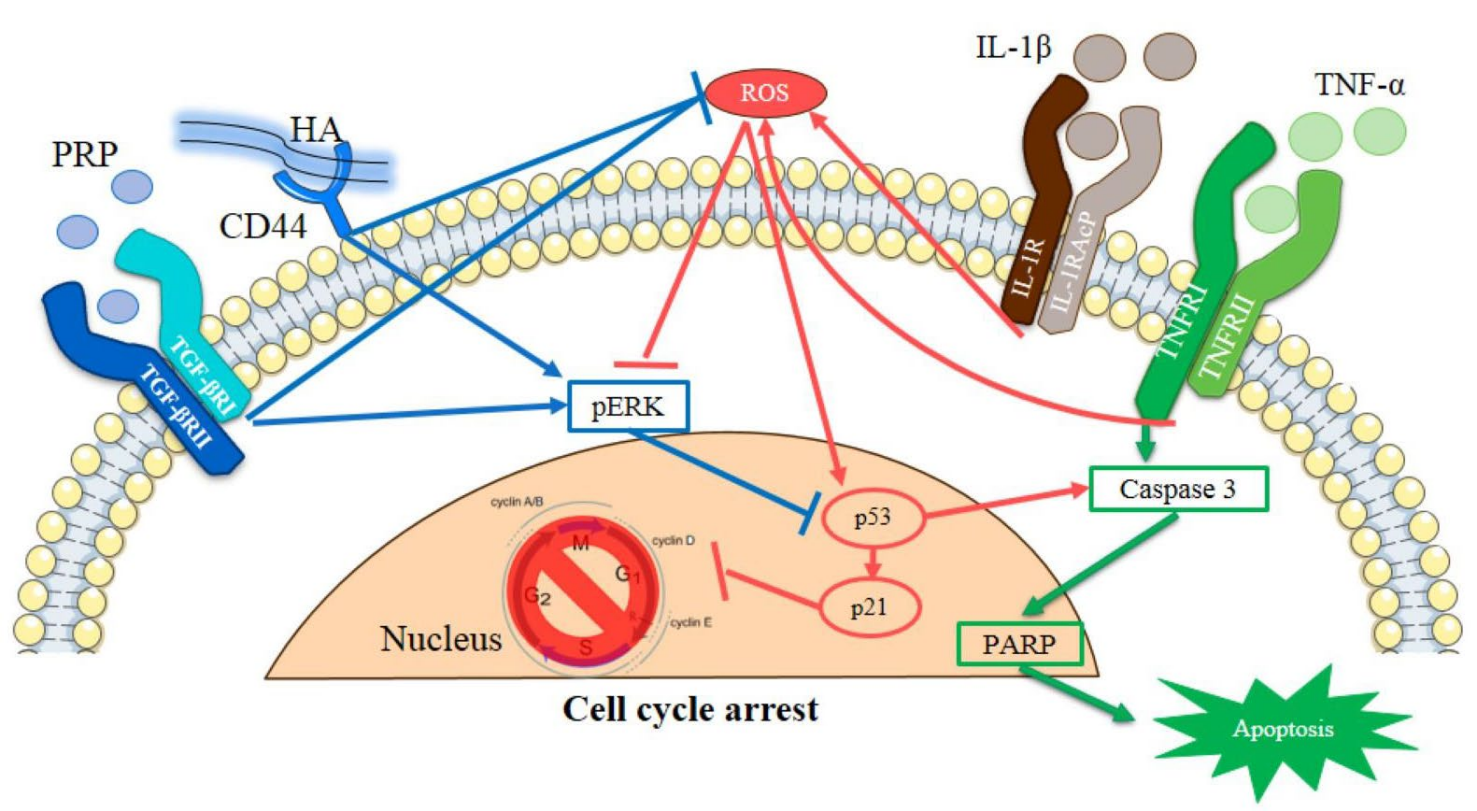
Schematic representation of HA and PRP-mediated cellular apoptosis in osteoarthritic chondrocytes.

Conclusively, in this *in vitro* OA model, the IL-1β+TNF-α (I+T) enhanced the production of ROS leading to cellular apoptosis of cleaved caspase 3, cleaved PARP, p53 and p21 proteins at molecular levels. Compared to HA or PRP, the combined HA+PRP could effectively inhibited ROS, increased anti-oxidant defense and activated ERK pathway, thereby inhibited p53 activity, resulting in prevention of apoptotic events (Fig. 7). It further retarded cartilage degradation and calcification through inhibiting the activity of MMP-1. Taken together, this study demonstrated the mechanism of anti-apoptotic effect of HA+PRP on arthritic chondrocytes. This imply that a combined HA+ PRP administration may be an effective therapeutic agent against clinical osteoarthritic pathology.

## MATERIALS AND METHODS

### Cell culture

Human chondrocytes cultures were obtained from 5 the patients who were undergone joint replacement therapy (age: 65, 68, 76, 79 and 86 years-old). An informed consent was obtained from the patients and all the protocols were followed as per of the Institutional Review Board (IRB. No. 201305026). First, osteoarthritic cartilage harvested from pateints was turned into pieces and digested with an enzymatic solution consisting of 8mg/ml hyaluronidase (Sigma), 8mg/ml collagenase (Sigma) and 2.5 mg/ml trypsin (Sigma) for 6 hrs at 37^0^ C. The cell suspension was centrifuged at 1500 rpm for 5 min and resuspended into DMEM/F12 (Gibco BRL) with 10% FBS (FBS, Gibco BRL), and 1% PSA (Biological Industries) in a humidified atmosphere containing 5% CO_2_ and passaged once a week. Thereafter, the OA chondrocytes were cultured, passaged and maintained in the same medium.

The IL-1β and TNF-α have been reported to inhibit chondrocytes proliferations and induce matrix degradations and in cartilage [59,60]. Therefore, we established *in vitro* OA model, by supplementing IL-1β (20ng/ml) and TNF-α (40 ng/ml) in the culture medium containing chondrocytes (passage 1), as described previously [21].

### Platelet-rich plasma and hyaluronic acid preparations

The preparation and quantification of PRP was followed as described in a previous study [61]. Various PRP concentrations according to ELISA results of TGF-β1 were dissolved in DMEM/F12 1% FBS medium. The HA of average molecular weight (MW) of 50-120 kD (Artzdispo, Seikagaku Inc., Japan) was prepared for HA conditioned medium in DMEM/F12 with 1% FBS medium for cellular maintenance.

### Cell viability and proliferation assay

MTT assay kit (Roche) was employed to assess cell viability using cell proliferation. Chondrocytes were seeded into 96-well plate at a density of 2×10^4^ cells/ml and treated with various concentrations of HA (250 µg/ml) or PRP (TGF-β1=1ng/ml)-conditioned medium, while 1% FBS was used as experimental control. The O.D. values were noted through MULTISKAN PC (Thermo Labsystem). Further, after the 2-day pro-inflammatory cytokine treatment, the HA, PRP or HA+PRP was employed to assess their effects of on cellular proliferation and the cell numbers were evaluated with cell countess (Life Technologies).

### Proteins extractions and western blot

Cell lysis was in RIPA buffer (50 mM-Tris, 150 mM NaCl, 0.5% DOC, 1% NP-40, and 0.1% SDS), thereafter, the 10µg of total protein was extracted, denatured for 5 min at 95 °C and separated on a 10% SDS-PAGE gel. Further, the proteins were transferred on to the PVDF membrane, and blocked with 4% BSA blocking-buffer. The membrane was then reacted with PARP (cell signalling, 9541, 1:1000), caspase 3 (cell signalling, 9661, 1:1000), p53 (cell signalling, 25275, 1:1000), p21 (cell signalling, 2947P, 1:1000), pErk (GeneTex, GTX61126, 1:1000), cyclin B1 (cell signaling, 4138P, 1:1000), cyclin D1 (cell signaling, 29785, 1:1000), cyclin E2 (cell signaling, 41325, 1:1000) polyclonal antibodies. Membranes were then incubated with anti-rabbit secondary peroxidase-conjugated antibody (17074P2, 1:5000). Additionally, the membranes reacted with β-actin (7074P2, 1:5000, Millipore) monoclonal antibodies were then incubated with anti-rabbit secondary peroxidase-conjugated antibody (7074P2, 1:5000). The bands were visualized by hyperfilm (Amersham Pharmacia) using the ECL plus-kit (Millipore Corporation) and images were analyzed using Mutigel-21.

### Flow cytometric analysis of Sub-G1 population

Chondrocytes were seeded into 6-well plate at a density of 3 x 10^5^ cells and cultured overnight. Thereafter, the chondrocyte cultures were replaced with conditional medium the treatment groups, including I+T, HA, PRP, and HA+PRP-treated group. After 48 hours, the cell layer was trypsinized and washed with PBS and fixed with 75% ethanol. 500 μL of RNase A (0.2 mg/ml, Sigma, 10109142001) and 500 μL of propidium iodide (0.02 mg/ml, Sigma, 11348639001) were added to the cell suspensions and the mixtures were incubated for 30 min in dark. The samples were then analyzed with flow cytometer (BD FACS Calibur. The number of cells analyzed for each sample was 10,000.

### Determination of Reactive oxygen species

Chondrocyte cell culture was collected and centrifuged at 10,000 g for 5 min. The supernatant was used to assay the in vitro concentration of ROS using OxiSelect™ In Vitro ROS/RNS Assay Kit (Cell Biolabs, USA, SAT-347). The level of intracellular ROS generation was detected using 2’, 7’-dichlorofluorescein diacetate (DCFH-DA). 50 μL of sample and 50 μL of catalyst was added to each well a 96-well plate, and after mixing well, incubated for 5 minutes at room temperature. Then, the 100 μL of DCFH solution was added to each well. Cover the plate reaction wells to protect them from light and incubate at room temperature for 15-45 minutes. The fluorescence was measure with a fluorescence plate reader at 480 nm excitation / 530 nm emission.

### Antioxidant assay

We employed the antioxidant assay kit (Cayman chemical, Cat No.709001, USA) to determine the level of total antioxidant capacity in the chondrocytes according to manufacturer’s instructions.

### Anterior cruciate ligament transection operations OA animal models

All the animal care and used protocols were approved by the Institutional Animal Care and Use Committee (IUCUC). In 8-week-old female C57BL/6J mice, the experimental OA was induced through transection of anterior cruciate ligament (ACLT) in the right knee, while sham operated mice were referred as the control. After 1 month of ACLT-induced OA, the HA, PRP, and HA+PRP were administered into right knee of mice (n= 6/group). After another 1 month, the animals were euthanized and the knee joints from all the groups were then harvested for histologic and immunohistochemical studies.

### Detection of chondrocytic apoptosis

The chondrocytes containing fragmented DNA were assessed in paraffin sections using a fluorometric terminal deoxynucleotidyl transferase dUTP nick end labeling (TUNEL) kit (Promega, USA, G3250), as per the manufacturer's protocol.

### Histologic and Immunohistological staining

Immunohistochemical staining was conducted on fixed tissue sections using avidin-biotin peroxidase technique. Briefly, unstained sections were deparaffinized with xylene and rehydrated with decreasing concentrations of ethanol. Non-specific binding was blocked with 4% bovine serum albumin (BSA). Avidin and biotin binding sites contained in the tissue samples were blocked using a commercial avidin-biotin blocking kit (Vector laboratories). Sections were then incubated for 30 minutes at room temperature with the following mouse anti-human monoclonal antibodies diluted in phosphate buffered saline (PBS) containing BSA; anti- MMP-1 (1:40 dilution) and incubated at 4°C for overnight (Calbiochem, Oncogene Science Inc. Cambridge). Sections were washed in ice-cold saline and incubated with a secondary biotinylated anti-mouse IgG. The activity of endogenous peroxidase was blocked using 0.3% H_2_O_2_ in horseradish peroxidase (Vector laboratories). Peroxidase activity was visualized using diaminobenzidine (DAB, Vector laboratories). This technique uses unlabelled primary antibody, biotinylated secondary antibody, and a preformed avidin and biotinylated horseradish peroxidase macromolecular complex. The slides were further rinsed in water and lightly counterstained with haematoxylin. Besides, knee-joints tissue sections were also stained with alizarin red S to determine the hypertrophic chondrocytes, indicating mineralization status of the extracellular matrix.

### Statistical analysis

Data are represented as mean ± SD for each group. The experiments were performed in the triplicates, and the differences between groups were estimated *student’s t-test* (Sigma Plot Version 10.0). Symbols with *, ** and *** indicate p< 0.05, p< 0.01 and 0.001, respectively.
